# Hay Fever

**Published:** 1878-10

**Authors:** 


HAY FEVER.—(*White Mountain Echo*, Sept. 7.) At a meeting of the United States Hay Fever Association, (composed of hay fever victims,) held the last Monday in August, at Bethlehem, N. H., a letter was received from Dr. R. Woodward, of Worcester, Mass., which disproves the hypothesis that the disease is essentially due to the effect of the pollen of plants upon the mucous membranes of the air-passages, and lends support to Dr. George M. Beard's theory that it is a disease of the nervous system. Dr. Woodward kept the pollen of rag-weed, Indian corn, golden rod, and other plants accused of producing hay fever, in his study, upon his table and scattered over his books and papers from the 8th to the 20th of August; but no symptoms of the affection were developed until the latter date. He says: "I have worked at my microscope for the last four weeks almost every day, and have examined the pollen of nearly one hundred plants, of all classes and kinds, and yet have not had a symptom till the appointed day came." He concludes that the disease is in him; that no irritation will develop it until the appointed time; but that after that time has arrived irritants of different kinds will excite and aggravate its symptoms. They are not, however, to be set down as the cause.

